# Quantitative Evaluation of Fundus Autofluorescence in Laser Photocoagulation Scars for Diabetic Retinopathy: Conventional vs. Short-Pulse Laser

**DOI:** 10.3390/life13091901

**Published:** 2023-09-12

**Authors:** Toshiya Kimura, Shuntaro Ogura, Tsutomu Yasukawa, Miho Nozaki

**Affiliations:** 1 Department of Ophthalmology, Laser Eye Center, Nagoya City University East Medical Center, Nagoya 464-8547, Japan; 2 Department of Ophthalmology and Visual Science, Nagoya City University Graduate School of Medical Sciences, Nagoya 467-8601, Japan

**Keywords:** diabetic retinopathy, panretinal photocoagulation, conventional laser, short-pulse laser, fundus autofluorescence, hypoautofluorescence

## Abstract

Short-pulse laser is popular for its advantages like less pain. However, its effectiveness is still debated. The aim of this study was to compare fundus autofluorescence (FAF) luminosity changes of laser photocoagulation scars between the conventional laser (0.2 s) and the short-pulse laser (0.02 s) for diabetic retinopathy. Conventional and short-pulse laser photocoagulations were performed in six and seven eyes, respectively. FAF images were captured at 1, 3, 6, 12, and 18 months after the treatments. To evaluate FAF, individual gray-scale values of the laser scars adjacent to the retinal arcade vessels were recorded; then, the mean gray values of the scars were divided by the luminosity of arcade vein. The average luminosity ratio of laser scars at 1, 3, 6, 12, and 18 months were 1.51 ± 0.17, 1.26 ± 0.07, 1.21 ± 0.03, 0.95 ± 0.11, and 0.89 ± 0.05 with conventional laser and 1.91 ± 0.13, 1.50 ± 0.15, 1.26 ± 0.08, 1.18 ± 0.06, and 0.97 ± 0.04 with short-pulse laser, respectively. Findings suggest the short-pulse laser displayed delayed hypoautofluorescence progression. This implies potential postponement in post-irradiation atrophic changes, as well as metabolic amelioration delay in the ischemic retina, when compared to conventional laser treatment.

## 1. Introduction

Diabetes, a metabolic disorder characterized by persistent hyperglycemia, poses a significant threat to various organs, including the eye, if not properly controlled. Over the past two decades, the prevalence of diabetes has steadily increased, with an estimated 422 million individuals aged 18 and above affected by this condition. Alarmingly, 35% of these individuals with diabetes also experience diabetic retinopathy (DR), which is the primary cause of vision loss in working-age populations [1,2].

While anti-vascular endothelial growth factor drugs have seen a recent surge in combating DR, laser photocoagulation has been the standard treatment for sight-threatening DR for several decades and continues to be a valuable therapeutic approach [3,4]. Panretinal photocoagulation (PRP) is employed to treat severe non-proliferative diabetic retinopathy (NPDR) and proliferative retinopathy (PDR) [5]. However, extensive damage resulting from PRP treatment can lead to complications such as macular edema, night blindness, visual field disorders, and reduced contrast sensitivity [6,7,8].

To address these challenges, a system called the short-pulse pattern scan laser system (PASCAL^®^ Streamline, Topcon Medical Laser systems, Santa Clara, CA, USA) was developed in 2006 [9,10]. This semi-automated photocoagulator delivers a pattern array of multiple burns in a rapid predetermined sequence. The advantages of this system include reduced heat production due to its short-pulse duration, resulting in minimal thermal damage and decreased eye discomfort compared to conventional laser treatments [10,11]. Furthermore, studies have reported that short-pulse lasers induce significantly fewer inflammatory cytokines and less macular thickening in patients with diabetic retinopathy [7,12]. However, it should be noted that short-pulse laser treatment of PRP may be less effective than conventional laser treatment in regressing retinal neovascularization in eyes with high-risk PDR [13]. Consequently, short-pulse lasers require a substantial increase in additional coagulation density and retinal burn area compared to conventional lasers to treat and completely regress deteriorating high-risk PDR [14]. The disparity in effectiveness can be attributed to the fact that the total burn area of PRP scars generated by conventional lasers is significantly larger than that of short-pulse lasers when an equivalent number of laser spots are employed [13,15]. Notably, the photocoagulation scars created by conventional lasers tend to expand over time [13,16,17,18,19,20], while scars from short-pulse lasers also undergo expansion, albeit at a lower ratio than those from conventional lasers [11,18,20,21].

To assess laser scars, various devices such as color fundus photographs, fluorescein angiograms, infrared images, and optical coherence tomography (OCT) examinations have proven to be useful [11,18,20,21,22]. Recently, our study compared the expansion rates of laser photocoagulation scars between conventional lasers and short-pulse lasers using fundus autofluorescence (FAF) [21]. FAF imaging is a noninvasive objective tool that evaluates macular and choroidal diseases, including age-related macular degeneration and retinitis pigmentosa [23,24]. It reflects lipofuscin accumulation in retinal pigment epithelial (RPE) cells, and a decreased signal indicates RPE degeneration [9,24,25]. As retinal laser photocoagulation primarily targets the RPE, monitoring FAF after laser photocoagulation can serve as an effective method to evaluate the residual RPE function and the efficacy of laser treatment. Our previous report comparing the two treatments, short-pulse and conventional lasers, indicated a difference in the hypofluorescence of autofluorescence in the coagulation spots between the two groups [21].

In this study, our objective is to compare the changes in FAF luminosity of retinal photocoagulated spots over time between conventional laser (0.2 s) and short-pulse laser (0.02 s) treatments for diabetic retinopathy. By conducting this study, we aim to evaluate the differences between these two devices and gain insights into their respective efficacy in treating diabetic retinopathy.

## 2. Materials and Methods

### 2.1. Study Design

This study is a retrospective cohort study conducted in accordance with the ethical standards stated in the 1964 Declaration of Helsinki. The study was approved by the Institutional Review Board of the Nagoya City University Graduate School of Medicine. Patients treated at Nagoya City University Hospital since September 2013, who were able to follow up for at least 18 months after laser photocoagulation for PRP, were enrolled in the study. Patients with media opacities such as corneal opacity, cataract, or vitreous hemorrhage that might affect FAF images were excluded. However, eyes with good FAF image quality with focal peripheral cortical lens opacity were not excluded.

All patients underwent a comprehensive ophthalmic examination, including fluorescein angiography, fundus photography, and FAF. Furthermore, an assessment was conducted to compare the laser conditions and the clinical outcomes.

### 2.2. FAF Aquisition

FAF images and color fundus photographs were acquired using either Optos^®^200Tx or Optos^®^ California imaging systems (Optos, Dunfermline, Scotland, UK) at specific time intervals: 1, 3, 6, 12, and 18 months post treatment. The acquisition and analysis of FAF images were performed by a single investigator (TK) to ensure consistency and minimize interobserver variability.

**Figure 1:**
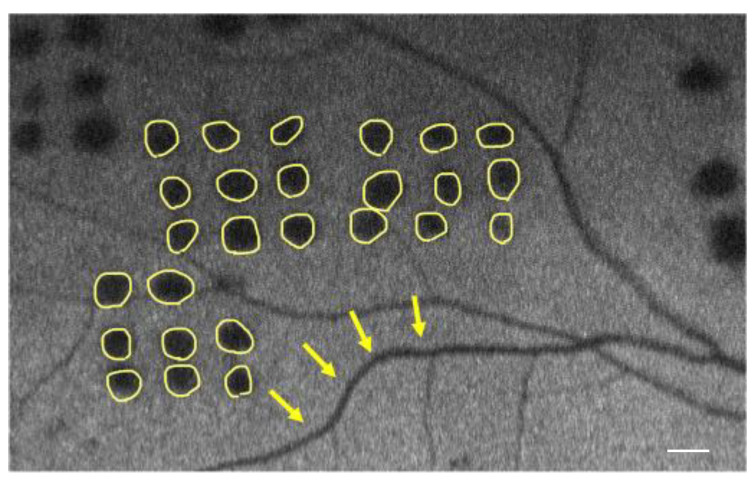
Representative image showing fundus autofluorescence (FAF) from the eye treated with a short-pulse laser; 18 months after treatment. The autofluorescence luminosity of the coagulated scars near the retinal vascular arcade (indicated by yellow circles) and the adjacent arcade vein (indicated by a yellow arrow) was evaluated and quantified by calculating the ratio of the mean gray value (Scale bar = 50 μm).

To quantitatively evaluate the FAF, the luminosity of autofluorescence in the coagulated scars was measured at each time point. The measurements were conducted using ImageJ software 1.53K (National Institutes of Health, Bethesda, MD, USA). Specifically, the average gray value of autofluorescence in the coagulation spots adjacent to the retinal vascular arcade was compared to that of the arcade vessels (veins). This analysis allowed us to calculate the ratio of autofluorescence intensity, providing a reliable indicator of the changes occurring in the treated areas (Figure 1). Notably, when the mean gray value of laser scars reached a value of 1, the scars exhibited hypoautofluorescence similar to that of the adjacent vein. The scale bar was determined based on measurements using Optos Advance.

Consequently, a comprehensive comparison of the resulting scars from both the conventional laser and short-pulse laser treatments was carried out, considering the quantitative FAF measurements and their corresponding time points.

### 2.3. Statistics

All data are presented as mean ± standard deviation. To examine potential associations, the Fisher’s exact test was utilized to analyze differences in gender, severity of DR, and history of cataract surgery. Additionally, a Student’s *t*-test was employed to compare the mean ages between the groups. The Mann–Whitney U test was conducted to assess the differences in the number of PRP shots administered. Furthermore, the incidence of additional laser treatment and the occurrence of vitreous hemorrhage were compared between the two groups using a Chi-square test. To analyze the luminosity rates obtained from the FAF measurements, the Bonferroni–Dunn method was applied. This statistical technique enabled the identification of any significant differences between specific time points within each group. A *p*-value of less than 0.05 was considered statistically significant.

## 3. Results

### 3.1. Patient Characteristics

Thirteen eyes (eight patients) with either severe NPDR (*n* = 10) or PDR (*n* = 3) were included. Six eyes were treated with the conventional laser, and seven eyes were treated with the short-pulse laser. The clinical characteristics of the patients are shown in Table 1. The conventional laser group included two PDR eyes, and the short-pulse laser group included one PDR eye. The conventional laser group also included one pseudophakic eye, while all eyes in the short-pulse laser group were phakic. The mean ages of the patients were 67.8 ± 7.6 years (range: 56–79) in the conventional laser group and 61.6 ± 10.4 years (range: 46–72) in the short-pulse laser group. There were no statistically significant differences in the patients’ characteristic parameters between the two groups.

### 3.2. Laser Setting Parameters

The laser treatment was performed with the conventional laser (Novus Varia, Lumenis, Santa Clara, CA, USA) or the short-pulse laser (PASCAL^®^ Streamline) using a yellow wavelength (577 nm). Both laser methods were applied with the same spot size (200 μm) but at different power levels to achieve similar grayish-white coagulation spots, utilizing the Ocular Mainster PRP 165 contact lens (Ocular Instruments Inc., Bellevue, WA, USA). For all patients, the laser power at which a light grayish-white color change was observed in the irradiated retina immediately after irradiation was used for treatment. The summary of the settings used for the conventional laser and the short-pulse laser is shown in Table 2. The mean number of laser shots performed was 1124 ± 303 spots in the conventional laser group and 3757 ± 693 spots in the short-pulse laser group. A statistically significant difference was observed in the total number of laser shots (*p* < 0.05, assessed using the Mann–Whitney U test).

### 3.3. Clinical Outcomes

In the conventional laser group, four eyes (67%) required additional laser treatment due to areas of nonperfusion and residual neovascularization at 6, 8, and 10 months post treatment. One eye (17%) experienced visually threatening macular edema, which was confirmed 4 months after PRP treatment, and required focal laser photocoagulation using the NAVILAS laser system (OD-OS GmbH, Teltow, Germany).

In the short-pulse laser group, two eyes (29%) needed additional laser treatment due to residual nonperfusion areas, ranging from 6 to 16 months after the initial treatment. During the 18-month follow-up after laser treatment, it was observed that one eye in the short-pulse laser group experienced vitreous hemorrhage, which originated from a retinal break rather than neovascularization. The incidence of additional treatment or vitreous hemorrhage did not show statistically significant differences between the two groups.

### 3.4. Luminosity Ratio of the Photocoagulation Scars

**Figure 2:**
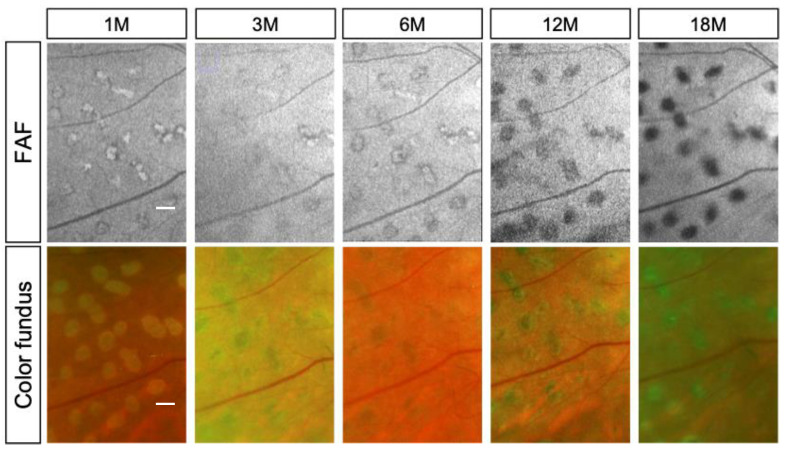
Representative fundus autofluorescence (FAF) images (**top**) and color fundus photographs (**bottom**) from the conventional laser group. The images were captured at 1, 3, 6, 12, and 18 months after treatment. At 1 month, FAF images show pronounced hyper-autofluorescence, which gradually transitions to hypofluorescence at 12 months and further hypofluorescence at 18 months post coagulation. Conversely, these changes are less evident in the corresponding color fundus photographs (scale bar = 500 μm).

**Figure 3:**
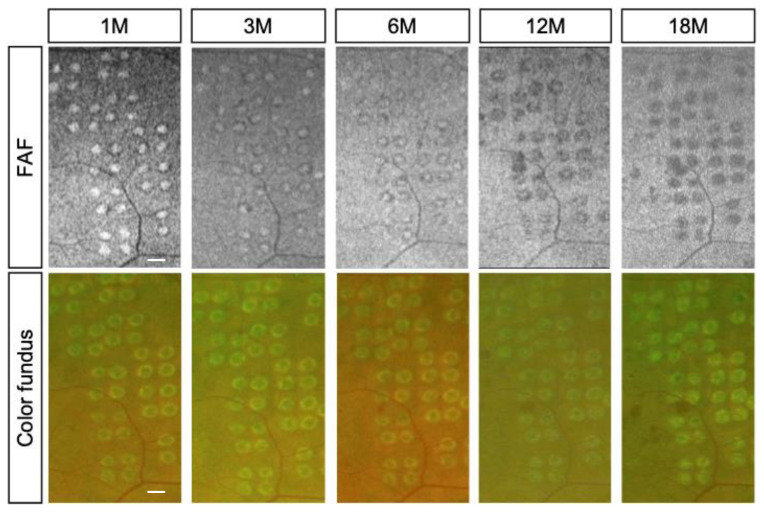
Representative images of fundus autofluorescence (FAF) (**top**) and color fundus photographs (**bottom**) from the short-pulse laser group. The images were captured at 1, 3, 6, 12, and 18 months after photocoagulation. Notably, the ring-shaped hypoautofluorescence surrounding the lesions demonstrates a gradual reduction over time. In contrast, detailed changes in the coagulation spots are challenging to discern on color fundus photographs, which is consistent with findings in the conventional laser-treated eyes (scale bar = 500 μm).

**Figure 4:**
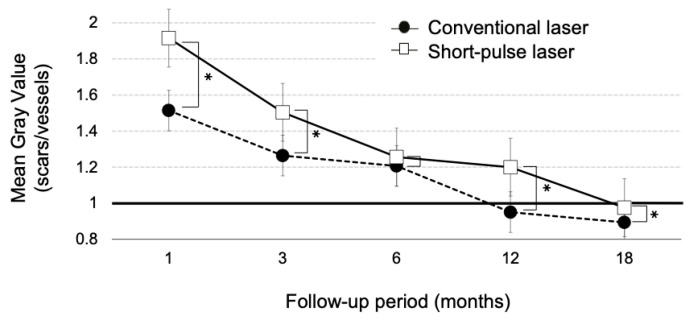
The graph illustrates the changes in the mean gray value of laser scars treated with the conventional laser (represented by closed circles) and short-pulse laser (represented by open squares) at 1, 3, 6, 12, and 18 months following laser treatment. The data shown here are from all eyes analyzed (*n* = 13). Notably, when the mean gray value of laser scars reached a value of 1, the scars exhibited hypoautofluorescence similar to that of the adjacent vein. Interestingly, the time point at which scar autofluorescence transitions to hypofluorescence is significantly delayed by the short-pulse laser spot compared to the conventional coagulation, and this difference remained statistically significant even after 18 months (* *p* < 0.05, Bonferroni–Dunn method).

Typically, laser scars appear as hyperfluorescent spots shortly after irradiation and progressively transition to hypofluorescence. For quantitative evaluation of FAF, individual gray-scale values of the laser scars adjacent to the retinal arcade vessels were recorded at each visit (Figure 2 and Figure 3). Then, the mean gray values of the scars were divided by the luminosity of arcade vessels (veins) using NIH ImageJ software. The luminosity ratio of scars with the conventional laser at 1, 3, 6, 12, and 18 months post treatment were 1.51 ± 0.17, 1.26 ± 0.07, 1.21 ± 0.03, 0.95 ± 0.11, and 0.89 ± 0.05, respectively. On the other hand, the luminosity rates of scars with the short-pulse laser were 1.91 ± 0.13, 1.50 ± 0.15, 1.26 ± 0.08, 1.18 ± 0.06, and 0.97 ± 0.04, at 1, 3, 6, 12, and 18 months post treatment, respectively. There was a statistically significant difference in the luminosity ratio between the two groups at 1, 3, 12, and 18 months (*p* < 0.05) after laser treatment. However, at 6 months post treatment, no statistically significant difference was observed between the two groups. (Figure 4).

**Figure 5:**
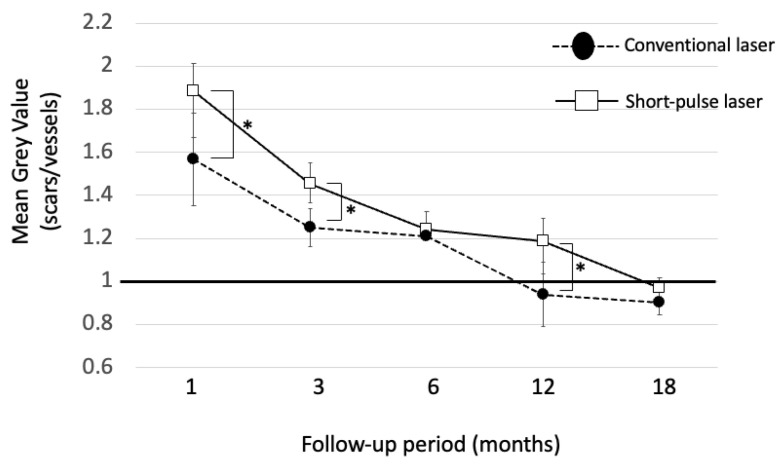
The graph displays changes in the mean gray value of laser scars in NPDR eyes treated with conventional laser (closed circles, *n* = 4) and short-pulse laser (open squares, *n* = 6) over 18 months. Both conventional and short-pulse laser treatments had small sample sizes. Short-pulse laser demonstrated a delayed transition to hypoautofluorescence compared to conventional laser, and a similar time course change was observed, as shown in Figure 4 with NPDR and PDR eyes. However, at 18 months after treatment, no significant difference was observed (* *p* < 0.05, Bonferroni-Dunn method).

To determine whether the effect was different in the eyes with NPDR or PDR, the ratio of mean grey value was also analyzed. Due to the small number, especially in PDR (*n* = 3), the results from conventional laser and short-pulse laser were mixed. As shown in Table 3, there was no difference in the ratio of mean grey value between NPDR and PDR. Then, we focused on the eyes with NPDR and compared the ratio of mean grey value between conventional laser and short-pulse laser. Both conventional (*n* = 4) and short-pulse laser treatments (*n* = 6) had small sample sizes. Short-pulse laser demonstrated a delayed transition to hypoautofluorescence compared to conventional laser, and a similar time course change was observed, as shown in Figure 4 with NPDR and PDR eyes. However, at 18 months after treatment, no significant difference was observed (Figure 5).

## 4. Discussion

For PRP, laser photocoagulation targets the RPE and adjacent photoreceptors while sparing the inner retina [26,27,28]. This process involves the destruction of metabolically active photoreceptors, leading to improved intraretinal oxygen delivery through enhanced oxygen diffusion from the choroid. Consequently, it mitigates hypoxia associated with retinal capillary loss, facilitating the regression of neovascularization [29,30,31]. However, the use of conventional laser PRP results in extensive destruction of the peripheral outer retinal layers, leading to complications such as visual field constriction, night blindness, and reduced retinal sensitivity [6,8]. These complications significantly diminish the patient’s visual quality. To address this issue, an alternative has emerged: PRP utilizing a short-pulse laser. This approach preserves retinal sensitivity, prevents the worsening of subjective symptoms in patients [18], and is becoming recognized as an alternative solution. Our findings provide robust evidence indicating that scars treated with the short-pulse laser exhibit a delayed transition from hyperautofluorescence to hypoautofluorescence compared to scars treated with the conventional laser. Importantly, the short-pulse laser scars remain luminous even at 18 months post treatment, suggesting a delayed progression of retinochoroidal atrophy when utilizing the short-pulse laser.

The question arises as to why the FAF signal exhibits a delayed transition to hypo-autofluorescence, indicating delayed atrophic changes in the short-pulse laser-treated groups. Short-pulse lasers require more power than conventional lasers to produce ophthalmoscopically visible spots [9]. However, due to their short irradiation time, the total energy (power × irradiation time) is lower for short-pulse lasers compared to conventional lasers. The cumulative pulse energy increases with pulse duration, indicating significant heat diffusion from the laser spot at conventional laser settings. Another indication of significant heat diffusion is the tendency for the creation of less localized and less homogeneous lesions with conventional laser settings [9]. Muqit et al. also demonstrated that the short-pulse laser treatment involves improved tissue oxygenation, RPE proliferation, and an infiltration of photoreceptors, while longer-duration laser treatment resulted in increased spatial oxygenation but also greater collateral tissue damage over time [32]. Notably, choroidal damage caused by the short-pulse laser is likely confined to the outer retina, whereas the conventional laser can lead to choriocapillaris destruction and subsequent RPE and photoreceptor atrophy [33], accelerating the hypoautofluorescence observed in FAF signals [34].

**Figure 6:**
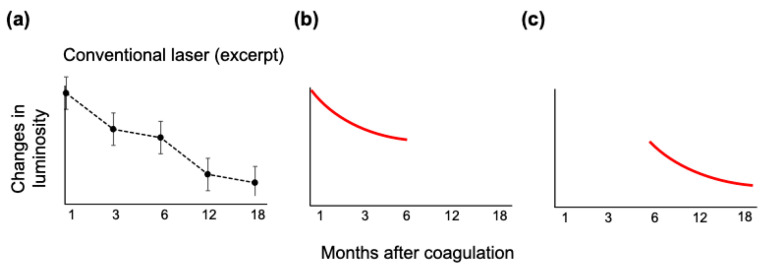
The figure illustrates the luminosity changes observed after coagulation with the conventional laser, providing insights into our speculative hypothesis. By examining the extracted graph from the conventional laser group (**a**), it can be observed that the graph can be divided into two distinct phases: 1–6 months (**b**) and 6–18 months (**c**). This division aligns with the autofluorescence (FAF) changes observed, wherein during the 1–6 months phase, autofluorescence transitions from hyperautofluorescence to hypoautofluorescence, forming a gentle downward convex curve. Moreover, during the 6–18 months phase, a distinctive feature emerges: a thickened hypoautofluorescence ring progressively encroaching towards the center. These findings are consistent with the FAF alterations observed during these respective time intervals.

Our study, along with previous research from other investigators, supports the concept that the FAF changes observed in conventional coagulation can be classified into two distinct phases: the 1–6 months phase and the 6–18 months phase [21,35]. During the initial 1–6 months phase, autofluorescence initially transitions into hyperautofluorescence and subsequently progresses gradually towards hypoautofluorescence, forming a gentle downward convex curve (Figure 6a,b). Framme et al. proposed that during this phase, the debris from damaged RPE cells and photoreceptors is phagocytosed by adjacent RPE cells or choroidal macrophages, resulting in the accumulation of phagosomes and storage granules that may contribute to the observed hyperautofluorescence [35].

In the subsequent 6–18 months phase, the hyperautofluorescence in the central area gradually transforms into hypoautofluorescence, creating a ring-shaped hyperautofluorescence surrounding the lesions, which also follows a downward convex curve (Figure 6c).

These observations suggest that the short-pulse laser treatment offers distinct advantages in terms of preserving RPE function and delaying the progression of atrophic changes, compared to the conventional laser approach. The FAF patterns observed in our study support the hypothesis that the short-pulse laser induces a more favorable healing response and minimizes collateral damage, contributing to the preservation of RPE integrity over time. These findings have important implications for the management of retinal diseases and further highlight the potential of short-pulse laser treatment as a promising therapeutic modality.

When comparing the size and expansion rates of laser scars, it was observed that scars produced with the conventional laser exhibited significant enlargement compared to those treated with the short-pulse laser, although both types of scars showed some degree of expansion [18,20,21]. This finding is further supported by a study that evaluated the scars using optical coherence tomography, which revealed RPE aggregation and photoreceptor cell atrophy up to 6 months after irradiation with the conventional coagulation. In contrast, scars did not extend to adjacent RPE cells, photoreceptor cells, or the choroid with the short-pulse coagulation, indicating that the damage extension was limited with the short-pulse laser, resulting in less scar expansion [36].

Several reports have demonstrated a significant reduction in retinal blood flow following PRP in eyes with PDR [37,38,39,40]. More recently, Iwase et al. reported that retinal blood flow was significantly reduced only during the 12 weeks following completion of PRP using conventional laser treatment but not with the short-pulse laser [41]. Although retinal blood flow decreased during PRP with both conventional and short-pulse coagulation, improvement in blood flow was observed only in the short-pulse coagulation group at 6 months after PRP, while no such improvement was seen with conventional coagulation [42]. Collectively, these findings suggest that short-pulse lasers may cause less injury compared to conventional lasers.

Clinical evidence indicates that a comparable number of photocoagulation treatments were insufficient to regress neovascular vessels in high-risk PDR eyes treated with short-pulse laser PRP, leading to an increased incidence of vitreous hemorrhage within 6 months after treatment [13]. The clinical limitations observed with short-pulsed laser treatment have often been attributed to the smaller enlargement of the laser scar. However, DRCR.net protocol S demonstrated that eyes treated with short-pulse laser PRP had higher rates of PDR progression (60%) compared to conventional single spot treatment (39%), regardless of the number of spots [43]. This result suggests that the nature of the scar itself may differ between short-pulse and conventional laser treatment, which is further supported by our study’s analysis of FAF luminosity. Furthermore, it is plausible that the effect of neovascularization suppression may require more time to manifest in eyes treated with the short-pulse laser.

Our study has several limitations that warrant consideration. Firstly, the small number of cases included in our study may restrict the generalizability of our findings. The limited sample size reduces the statistical power and may limit the ability to draw definitive conclusions. Therefore, caution should be exercised when extrapolating our results to a larger population. Secondly, our study design was retrospective, relying on existing data, which introduces the potential for inherent biases and confounding factors. Although we attempted to minimize these biases through rigorous data collection and analysis, the retrospective nature of the study design leaves room for uncertainties.

Additionally, despite comparing the short-pulse laser group with the conventional laser group, we did not observe any clear differences in clinical outcomes [21]. This lack of discernible differences could be attributed to several factors, including the relatively small number of PDR eyes enrolled in this study. Furthermore, the time course of hypoautofluorescence may exhibit variations between NPDR and PDR; however, the limited representation of PDR eyes may have affected the ability to detect significant differences between the two treatment groups. To enhance the robustness and validity of the findings, future studies should aim to include a larger number of PDR patients. To address these limitations and further advance the knowledge in this field, future research efforts should focus on increasing the number of patients with PDR to strengthen the statistical power and improve generalizability. Moreover, conducting combined studies with longer follow-up periods would provide valuable insights into the long-term effects and outcomes of the different laser treatments. Furthermore, the incorporation of additional imaging techniques such as OCT b-scan and FAF analysis of the laser scars could provide a more comprehensive assessment of the treatment effects and enable a deeper understanding of the underlying mechanisms.

In contrast, a notable trend in ophthalmic lasers is the ongoing development of less invasive techniques. Among these, subthreshold laser therapy has emerged as a promising approach for managing macular edema by selectively stimulating the RPE without causing visible tissue damage [44,45]. This non-destructive method aims to improve edema and restore visual function [46,47]. Additionally, recent advancements have led to the adoption of subthreshold PRP for non-proliferative diabetic retinopathy (NPDR) [48] and PDR [49]. Although several reports have highlighted its potential effectiveness, subthreshold PRP with confluent laser spots would take a lot of time; it would have to be conducted during several laser sessions, and the lack of large-scale clinical trials warrants cautious interpretation of these early findings [50].

If validated in future studies, subthreshold PRP could revolutionize the conventional understanding of PRP’s mechanism. Traditionally, PRP has involved the deliberate destruction of the outer retinal layer in ischemic regions to enhance oxygen supply to the inner retina and reduce VEGF levels. Gozawa et al. have shown that PRP suppresses the level of VEGF in the ischemic retina, supporting the mechanism by which PRP works [51]. However, subthreshold PRP proposes an alternative paradigm, employing subvisible laser energy to achieve therapeutic outcomes with reduced adverse effects.

However, presently, the implementation of subthreshold PRP remains limited, and prevailing guidelines advocate for classic PRP techniques utilizing either conventional laser or short-pulse laser [52]. Despite these challenges, our study has the potential to function as a biomarker for assessing the alteration in RPE and for speculating the efficacy of PRP treatments. Our study findings highlight the utility of FAF imaging in evaluating the changes in autofluorescence following laser photocoagulation. Scars treated with the short-pulse laser exhibited a delayed progression towards hypoautofluorescence compared to scars treated with the conventional laser. This suggests that the short-pulse laser may have a delayed effect on metabolic changes in RPE after PRP. Therefore, when using short-pulse laser therapy for diabetic retinopathy, including high-risk PDR, it is important to consider the treatment parameters to optimize its efficacy and outcomes. Overall, our study contributes to the understanding of the differences between short-pulse and conventional laser treatments and emphasizes the need for careful consideration in selecting appropriate laser therapies for diabetic retinopathy.

## 5. Conclusions

Our study demonstrates that monitoring FAF changes over time can serve as a useful indicator for determining the need for additional laser treatment when insufficient hypoautofluorescence changes are observed to prevent neovascularization.

## Figures and Tables

**Table 1:** Patient characteristics.

	Conventional Laser	Short-Pulse Laser	*p*
Number of eyes	6	7	
Mean age, years old	67.8 ± 7.6	61.6 ± 10.4	0.32 ^a^
Male: Femal	5:1	6:1	1.0 ^b^
NPDR: PDR	4:2	6:1	0.52 ^b^
Phakik eyes: pseudophakic eyes	5:1	7:0	1.0 ^b^

NPDR, non-proliferative diabetic retinopathy; PDR, proliferative diabetic retinopathy. ^a^ Student’s *t*-test, ^b^ Fisher’s exact test

**Table 2:** Settings of the laser treatments.

	Conventional Laser	Short-Pulse Laser
Spot size (μm)	200	200
Spacing (spot)	1	0.75
Pulse duration (ms)	200	20
Power (mW)	100–269	300–500
Wavelength (nm)	577	577
Mean number of total PRP shots	1124 ± 303	3757 ± 693

PRP, panretinal photocoagulation.

**Table 3:** The ratio of mean gray value (scars/vessels) between NPDR and PDR.

	1 Month	3 Months	6 Months	12 Months	18 Months
NPDR (*n* = 10)	1.76 ± 0.21	1.37± 0.13	1.23 ± 0.06	1.09 ± 0.16	0.94 ± 0.06
PDR (*n* = 3)	1.64 ± 0.34	1.46 ± 0.25	1.25 ± 0.08	1.07 ± 0.15	0.92 ± 0.06
*p*-value	0.26	0.23	0.35	0.46	0.30

Bonferroni–Dunn method. NPDR, non-proliferative diabetic retinopathy; PDR, proliferative diabetic retinopathy.

## Data Availability

Researchers can contact Miho Nozaki, (miho.nozaki@gmail.com) for details of the protocol and results.
